# Vaccination
*Conclusions Statistiques contre les détracteurs de la vaccine, précédés d'un essai sur la méthode statistique appliquée à l'étude de l'homme. Par le Dr. Bertillon. Paris: Victor Masson.Papers relating to the History and Practice of Vaccination (Letter to the President of the Board of Health). By John Simon, F.R.S. London: 1857.


**Published:** 1857-10

**Authors:** 


### VACCINATION.*

There is no element in the human mind more broadly marked than that love for change which, in its influence on general politics, is called the revolutionary tendency. The revolutionary tendency is not, however, confined to politics. In political matters, it is true, we see it in its most exaggerated form; but the idea is everywhere; it pervades science, it pervades history; sometimes it does good, and marks an era in wisdom; sometimes it does evil, and marks an era in folly; sometimes, when it plays its worst and most nonsensical parts, when it violates common sense and opposes fact, it is not without its uses. It sets up a reaction. The friends of order must sustain their cause, even though they be right; they had been lax before; they must be active now. They heap up evidence; they prove, instead of merely asserting, facts; and truth is benefited by the slur that has been cast upon her.

As a memorable instance of the revolutionary tendency in science, few examples of late years have created a greater sensation than the endeavour that has been made to uproot the discovery of Edward Jenner, and to prove that vaccination, instead of being a generally recognised blessing, should rather be received as a curse on humanity, and a disgrace to science. In the early days of vaccination, the points connected with its utility, or its evils, were discussed in what are called general terms. Individual men spoke according to their individual experience; and, taking it all in all, this individual experience stood out in favour of vaccination. In the face of prejudices, too, the people recognised its truth; for its great facts were self-evident, without any measurement or scientific symbol. Fifty years ago, let a man stand in a market, in a theatre, in any crowded assembly, and one out of every two or three of his neighbours bore on their faces the deep brand of the great epidemic. Jenner came, and the face of human nature changed. Year by year the pock-marked community died out, and their places were filled by less blemished physiognomies; and this has gone on until, in these days, the small-pox impression is well nigh out of print, and is no more called for again than the library of trash that has been written against vaccination.

A sort of simple common sense demonstration is certainly

---

* Conclusions Statistiques contre les détracteurs de la vaccine, précédés d'un essai sur la méthode statistique appliquée à l'étude de l'homme. Par le Dr. Bertillon. Paris: Victor Masson.

Papers relating to the History and Practice of Vaccination (Letter to the President of the Board of Health). By John Simon, F.R.S. London: 1857.

written in this single physiognomy-improvement fact; a demonstration which every body could see and appreciate: add to which every body (a few blockheads excepted, only to support the rule) felt, that when small-pox was at their doors, it was good to be vaccinated, and found that it *was* good because after it they did not die or suffer. So these and many other things combined, gave to vaccination a general recognition, and to Jenner an universal claim to distinction in biographical history.

Whether it be the fact that things generally believed in are not safe as beliefs until they become mathematically demonstrable, it is not necessary to discuss; but, as regards the vaccination question, it is to be noted that, when general assent had been accorded to it, it became subject to dissent by another line of argument, the statistical line, so called to distinguish it from the *fides populi.*

The statistical argument once brought into play against vaccination, discoveries began to rise like rockets, and fall like the sticks. Jenner's discovery, it is true, was never seriously shaken; but it had to be proved by the very arguments which were now used against it. Once assailed on mathematical principles, it must needs be defended on the same principles, or it must lose ground. To its defence in this line, the distinguished Bertillon put forth his best energies; and, in the book we now review, he chronicles some of his labours,

Anxious in the commencement of his work that his readers may understand him, Bertillon explains his ideas of the meaning of the statistical method of inquiry. On this subject there is great want of unanimity and soundness. Bertillon's explanations run, in brief, as follow.

The phenomena of nature are so complex, that without analysis they may always escape science; man living in a state of incessant contemplation before the magnificent unity of creation, would wear out sensation without arriving at knowledge. But analysis, in temporarily isolating an object, in concentrating on the object the attention dispersed on all, in permitting it to be submitted to all the proofs of which it is susceptible, enables one to study the object, and, through this study, to arrive at positive science.

The first method of analysis which naturally presented itself, was to consider each object separately, to submit it to the senses under all its states, and thus to study each property. This method has always sufficed when the extent of the object of study, as well as the intensity of its action, has been sufficient to affect the senses forcibly. Thus the first requirements were satisfied. But man soon met with a limit to his work through the imperfections of his organs. Things which escaped the naked eye, betrayed their existence in their effects; and the microscope came forward to render its wonderful services. Things which, out of the domain of optics, escaped observation, and which related to wide spread influences on man, geological, geographical, social, sexual, required also an instrument for their deeper analysis; the instrument was found in the science of great numbers, its name Statistics.

Having thus explained his views as to the meaning of statistical science, and dwelt at some length in the way of illustration, M. Bertillon exposes the opinions of those who are opposed to vaccination, and deals throughout in many hard blows against M. Carnot, a man who seems to have been fatally bitten by an antivaccination mania. In this battle, Bertillon is ably supported by Simon; and, as many of our readers may not know who M. Carnot is, or what his heresy, we may be excused for following our authors' narratives. Carnot was originally an artillery officer, and is described by Simon as having more zeal than judgment. He, Carnot, believes that he has discovered what he calls a displacement of mortality, and that, within the present century, deaths which used to occur in early infancy, now occur between the ages of fifteen and thirty. He alleges (we are quoting Mr. Simon *verbatim*) that the female death rate in Paris, for the ages of fifteen to twenty-five, has doubled in the last thirty-eight years; that the annual mortality of the French army on home service was 2 per cent. during the period of 1819-47, but had been only one per cent. before the Revolution of 1789; that of male and female deaths in Paris, at ages above fifteen, only 35 per cent. used to happen between the ages of fifteen and forty-five, and that now 50 per cent. are in this category; that the chances of an infant reaching forty-one years of age, are the same now as they were in the last century; that the death lists of Paris for 1840-9, compared with those of fifty years previously, show some diseases (typhus, cholera, dysentery, and colic) to have increased almost as much as others (small-pox, measles, convulsions, and croup) have declined; that the annual marriages in France exceed by twice as many as they forty years ago exceeded the marrying proportion ($\frac{4}{5}$) of females who annually (on Duvellard's estimate) reach the connubial time of life, and that this increase denotes a doubled annual number of second marriages, or, in other words, a doubled annual quantity of early widowhood; that, with a greatly increased number of marriages in France, there is a diminished number of births; that the births are rapidly tending to become less numerous than the deaths; that the depopulation of France is in imminent danger, which must begin to realise itself within the next few years; that gastro-intestinal disease—especially typhoid fever—is the agent of this destruction; and finally, that the cause of this complicated derangement is the practice of vaccination.

So much for M. Carnot. Mr. Simon thus summarily disposes of his labours. "Now first let the concluding word of the summary be criticised. M. Carnot's statistics allege a difference in adult vitality between the France of to-day and the France of the last century. Supposing the statistics to be correct, does he give any sufficient reason for ascribing to vaccination that deteriorated state of adult life which he professes to have discovered? So little does he this, that in any of the sentences where damnatory conclusions are drawn, if there were substituted at hazard, for his word, *vaccination*, the mention of any other historical event, belonging to about the same period of time as Jenner's discovery, M. Carnot's logic would scarcely suffer by the change, or his new conclusion be less warrantable than his first. *Post ergo propter* was never more whimsically illustrated. For the argument goes simply to claim as the effect of vaccination, whatever evils have happened since its discovery; and M. Carnot's moderation may be praised, that with the infinite resources of his proof, he did not also convict Jenner of causing the last year's inundations of the Rhone."

This quotation, if it did not in its last sentences flavour feebly of M. Carnot's own logic, might be considered as a scientific shutter-up of no mean force.

Bertillon, on his side, enters more fully into detail in opposition to the redoubtable Carnot. We think it useless to follow him in all his arguments. One or two examples will suffice to indicate on what false, dangerous, and ridiculous data Carnot's statistics are founded.

Bertillon shows that the statistics of Paris, on which Carnot bases his conclusions, are not reliable as data for any such assumption. Take, for example, the statistics derivable from the census of 1851. Here there are three leading errors. 1. Great numbers of newly born children are omitted. 2. There is a surcharge in the numbers of persons living at the ages of thirty, forty, forty-five, and fifty, to the detriment of the intermediate ages. 3. The age of twenty years has evidently been made the basis of a deception, executed without doubt in the hope of escaping conscription.

Again, Carnot has drawn his views from statistics referring to women only ; in order, so runs his sophistical explanation, that the deductions may be independent of any modifications in the male sex, arising from wars and other serious accidents. Bertillon asks, with pertinacious reason, whether M. Carnot does not also think that the unfavourable conditions under which women are placed in large towns, weigh much more heavily on them than on men ?

Once more, Bertillon argues, with common sense force, that the comparison of mortuary tables is not enough in itself to lead to a true appreciation of the mortality of each age. For example, in one thousand deaths of all ages, during the past century, we learn from Montyon or Duvillard, that there were about thirty-one deaths between the ages of twenty and thirty years ; while in our time, M. Heuschling has found seventy-five.

Might it not, therefore, be concluded from this fact, according to the ordinary method pursued by the opponents of vaccination, that adults succumb in much larger numbers in these days, than they did heretofore ? This would be to commit a gross error, since the increased mortality of adults may be distinctly traced to the circumstance that there are now living a greater number of adults, so that the proportions are the same; while, apart from this consideration, granting that the sum total of the living of each age remained stationary, we should not be authorised to conclude that adults succumb in greater numbers, because, out of a thousand deaths at all ages, we now find seventy-five deaths between twenty and thirty years, in the place of sixty-one deaths, as in times gone by. For, if the mortality of the other ages has diminished—and we know for a fact that the mortality of infancy has most notably decreased, while that from twenty to thirty years of age has remained stationary—it will result that, in the same number of deaths, there will be found a greater number between twenty and thirty years, since the deaths of the other ages will have lessened.

We need not follow out this opposition further. Our English statists have long since seen and felt the danger of incorrect deduction from statistics. An unscrupulous man may use them for any purpose. A scrupulous man uses them with fear and trembling. The study of statistics is as inductive in its purest phase as experiment ; but it is also as dangerous, in that it may be raised into the grossest empiricism. Statists, of all men, should look before they leap. Suffice it to say now, that Carnot and his followers have broken down entirely ; and that, if statistics prove nothing as yet, on safe grounds, in favour of vaccination, they most assuredly bring no charge whatever against it. Jenner's foot is on the statist's throat. What statistics really say on the question is very skilfully summed up by Mr. Simon.

"So far, then, as these populations" (the populations of Sweden, Denmark, and London) "are concerned, it appears that while under the influence of vaccination small-pox has been diminishing its ravages, so under other influences have other diseases been diminishing theirs. Under other influences, I say; for the causes of fever, the causes of cholera, the causes of consumption, are several and special causes. Each disease is affected for better or for worse by influences proper to itself; and the prevention of small-pox no more implies the prevention of fever, than to sow barley implies the reaping of wheat. But what has to be noticed (so far as these materials inform us) is, that annihilation of small-pox may be tried for as an unequalled physical good; that hitherto there is no trace of evidence that other diseases become more malignant in proportion as that one is subdued."

Having dealt with the statistical part of the question thus far, Mr. Simon fell back, in his defence of vaccination, on the argument of individual experience. He drew out a series of questions, which were sent for answer to numbers of eminent practitioners of medicine, to foreign governments, and the like. The questions ran as follow.

"1. Have you any doubt that successful vaccination confers on persons subject to its influence a very large exemption from attacks of small-pox, and almost absolute security against death by that disease?

"2. Have you any reason to believe or suspect that vaccinated persons, in being rendered less susceptible of small-pox, become more susceptible of any other infective disease or of phthisis, or that their health is in any other way disadvantageously affected?

"3. Have you any reason to believe or suspect (*a*) that lymph from a true Jennerian vesicle has ever been a vehicle of syphilitic, scrofulous, or other constitutional infection to the vaccinated person; or (*b*) that unintentional inoculation with some other disease, instead of the proposed vaccination, has occurred in the hands of a duly educated medical practitioner?

"4. Do you (assuming the provisions to exist for a skilful performance of the operation) recommend that, except for special reasons in individual cases, vaccination should be universally performed at early periods of life?"

The objections that may be urged against these questions by the opponents of vaccination, are, that the questions are leading ones, and are sure to elicit the required answers. We ourselves think that the results obtained would have been sounder if the method of questioning had been reversed; *i. e.* if the questioner had presumed for the moment that vaccination had its evils, and had asked his answerers how often they had seen such and such results. Nevertheless, there is no disputing the importance of the replies, or the fact that 539 medical men, all of eminence, have joined heart and voice in giving a favourable answer to the first three questions, and in supporting in full the discovery of Jenner as a protective, and its innocence as a practice.

Amongst some enthusiasts, the idea is prevalent that, by a perfect system of vaccination, small-pox might be entirely removed from the world. Is this extreme? The evidence collected by Mr. Simon is most interesting in reference to this topic. We would that we had the space to quote largely on this point from the extended replies met with in this report. We must be satisfied to pick out one representative fact. An extract from an explanatory paper accompanying the official answers from Denmark gives the following decisive particulars.

"Of the great epidemics of small-pox in Denmark, history mentions that of 1502, of 1556, that of 1716, and perhaps several others; but we search in vain for statistical returns exhibiting the number of individuals cut off by these epidemics. The disease raged year by year in the towns, as well as in the country; and although it attained a frightful height every fourth and seventh year, attended with typhoid fever, scarlet fever, and especially measles, yet our annalists did not feel themselves called upon to make any returns of an occurrence so common as this: the merits of the science of statistics, as applied to sanitary purposes, were at that time too little appreciated. In the face of such melancholy considerations, it is satisfactory to be able to report that this disease, since the universal introduction of vaccination (1810), has not only lost its worst sting, but that the disease has not shewn itself in Denmark for more than fifty years. The reporter states that the annals of Iceland give the history of the epidemic of small-pox occurring in that country from the year 1241 to the year of the great epidemic in 1707: it is said to have been brought into the country by some wearing apparel belonging to an Icelandic student, who fled from Copenhagen for fear of the small-pox, took that disease on board the vessel, died, and was buried in Norway. Of the then population of Iceland, somewhat exceeding 50,000, this disease carried off, according to reports, 18,000. In that country, where the parishes are so thinly populated, there were tombs in the churchyards in which thirty, thirty-four, to forty individuals were interred in one day. It was no unusual occurrence for persons having once gone through the disease, and bearing the marks upon them, to be attacked again and die. In 1785-86, and 1787, 1,425 died; this was the last time the disease occurred in that island during the last century.

"Since the introduction of vaccination, small-pox has only once occurred in Iceland, viz. in 1839, when it was brought to the northern division of the island. It was very mild, and was prevented from spreading to the southern division by measures of isolation. Several individuals were seized with the disease, who had it in 1785. Of the population of the town of Reikavick and its environs, amounting to from 1,200 to 1,300, of whom the greater part were vaccinated, only fifteen died. At a fishing cove, however, where only a few had been vaccinated, forty died out of a population of about 600. The disease continued to prevail in 1840. The population of Iceland, which in the twelfth and thirteenth centuries is said to have been 120,000, was 46,201 in 1769, and 57,094 in 1840."

The common as well as the statistical sense of mankind stamps these details with the seal of truth. Wherever vaccination is faithfully and scientifically practised it has prospered, we had almost said without an exception. Yet one exception is claimed even by its friends. Mr. Brown of Nepaul, who gives a most interesting account of the origin and success of vaccination in that Indian province, tells us, that inoculation is best suited for the inhabitants of Bengal, and that probably vaccination is too weak to act as a prophylactic in such individuals. Mr. Simon should seek an explanation of this statement. Is it based on fact and rigid experiment?

The study of the works here introduced to our readers opens up a number of important social, as well as scientific, questions. It is clear as crystal that vaccination is the preventive of small-pox, and that whole countries are saved from the scourge by its influence. But England, the birthplace of vaccination, is perhaps the least saved of all. We take up the Registrar-General's returns, and, during a period extending from 1838 to 1848, we find 462,227 deaths recorded as from these seven epidemical diseases, taken in order according to their fatality;—typhus, scarlet fever, measles, hooping cough, small-pox, influenza, and erysipelas. Small-pox, it is true, is fifth on the list; but it numbers out of the total of deaths 59,460 victims. The fact of such a sacrifice, in the presence of an antidote, is something in horror, that exceeds belief.

Why this sacrifice? facts against vaccination? No. Loose law in regard to the practice of it? No. What, then? Ignorance, prejudice, and the use of force for reason: the last most. Law and compulsion, in fact, are forces that will bring vaccination into entire disrepute in these islands. Law and compulsion are only serviceable to the Englishman when he knows, or thinks, or feels, that the law is right, and is for his protection. If you cannot convince him through his reason that a law is right, you will never beat it into him through his fear. On the contrary, the more you threaten, bully, and coerce, the more thoroughly will you satisfy him as to the correctness of his own views, and prevent him from inquiry altogether. What can be done with such an obstinate? does some one ask. Anything that is reasonable, is the reply. But drop the whip. An English community, that would not move for a shower of government commands, will move at once to a few sensible cadences rung on the alphabet. The point, therefore, is to instruct, prove, convince. What sensible man, poor or rich, cares to act without a clear view to utility?

We are advised by the preface to Mr. Simon's questions, that the late President of the Board of Health intended to bring in a new vaccination measure, or obtain a commission of inquiry; and as for that object the present Report was drawn up, there is a great deal in the intent to be thankful for. If, indeed, improvement in the system of vaccination be intended in the object, the intent is mainly carried out in the book here supplied. Let the Government of this country take but one other step. Let them have this report of Mr. Simon carefully and pleasantly condensed; let its facts be made easy, and its arguments simple; let it thus be (we must forge a word) primerified and then sown broadcast over the whole country, through the clergy and the Esculapian professors, so that every stupid may read or hear read what is the end and meaning of vaccination, and more will be done for vaccination than by a thousand acts or a million fines. As it is not probable that even the most common people enjoy small-pox, or have any other wish than to see the last of it, it is but logical that they would not have it, if they felt sure they could avoid it without risk. Make them sure, then; this is the political card.

Reviewing this question, indeed, from the true practical point, viz. a knowledge of the poorer classes, and the objections made to vaccination by them, we are prepared to say that it is not with the very ignorant that the objections originate, but amongst those who are most acute, who rank among their fellows as superior in argument, who read a little, think much, and possess special independency of will. These men (they are to be found bespread through every village and town community) have their reasons against vaccination, as fully as we have ours for it. Their reasons are false, but they are reasons; and, to their untutored observation, the reasons are all powerful. Their great argument is not against vaccination as a preventive of small-pox (at all events, not as a general rule), but against it as the propagator of other diseases. Nay, we wrong the poor in presuming that they are the only objectors on this score; for, whatever the facts may be, there is in the human mind some feeling so instinctively strong against the possible intermixture of health and disease, that we doubt whether the firmest advocate of vaccination would let his healthy babe be vaccinated from a sickly one, or would allow an operator to do his work from an unknown source, without inquiring first, and anxiously, whence the ivory point received its virus. Is this instinctive feeling wrong? Argument says yes; but experience finds it to be as strong as it is wrong; and, until this feeling can be appeased by fact and sound argument, and met by some plan that shall yield assurance of a healthy virus for every infant, the prejudice will be national, and all law ceremony null and void. Law-making becomes idiotcy when nature leads the opposition.

We cannot conclude, without expressing our deep thanks to Mr. Simon for his most laboured and invaluable report. By it the Board of Health has positively merited its twelve months reprieve from death; and next year it may, if it likes, renew its lease on the same terms. If it would not violate the principles of circumlocution too much, it would be useful to beg that a blue book might, for once, go through a second edition. In such second edition, the basis of the work might be widened. The experience of all the medical men in the United Kingdom, of all the medical officers of the services in all parts of the world, of all the great hospitals of the world, and of all the leading practitioners of all the great towns in the world, might be sought, obtained, and chronicled. The individual method of proof would thus by its vastness compete with the statistical; and, as the defence and support of vaccination rests naturally with England, this great work, the plan of which is already designed and in part carried out, remains in her hands for completion.

